# Accuracy and saving of time using a new algorithm for quantitative measurement of infarct and edema size in patients with acute myocardial infarction

**DOI:** 10.1186/1532-429X-16-S1-P199

**Published:** 2014-01-16

**Authors:** Gunnar Lund, Dennis Säring, Julia Cürlis, Dominik Barz, Maxim Avanesov, Enver Tahir, Kai Muellerleile, Gerhard Adam

**Affiliations:** 1 Dept. of Radiology, Hamburg, Germany; 2 Medizinische Informatik, Hamburg, Germany; 3 University Heart Center, Hamburg, Germany

## Background

Cardiac magnetic resonance imaging (CMR) enables infarct and edema size measurement on LGE- and T2w-CMR, respectively. However, evaluation of infarct and edema size using a threshold method is time consuming and relies on accurate placement of normal regions of interest and manual delineation of the respective areas. The purpose of the study was to analyze the accuracy and expenditure of time of a new algorithm for infarct and edema size measurement using LGE- and T2w-CMR in comparison to a standard evaluation strategy.

## Methods

CMR studies from 15 patients with acute myocardial infarction were quantitatively evaluated by 2 experienced and 2 novel, but trained observers in respect to infarct and edema size using a threshold method. For standard evaluation the signal intensities of remote myocardium were measured in five regions in remote normal myocardium. Images were thresholded to a level >2 SDs than that of normal myocardium and the respective areas were manually traced. The new evaluation algorithm simply required encircling of the infarcted/edematous areas and of remote normal myocardium. Subsequently, images were automatically thresholded and the areas were calculated according to a threshold >2 SDs. Evaluation times were recorded for both evaluation strategies.

## Results

Mean acute infarct size was 14 ± 10 %LV or 28 ± 25 gram using standard evaluation and 14 ± 9 %LV or 27 ± 24 gram for the new evaluation (P = ns). Edema size was 23 ± 11 %LV or 45 ± 30 gram using standard evaluation and 24 ± 12 %LV or 46 ± 29 gram for the new evaluation (P = ns). Agreement between the experienced and the novel observers was good for infarct size with 3.3 ± 11.3% for standard evaluation and 4.7 ± 12.0% for the new evaluation (P = ns). The agreement for edema size was 7.8 ± 15.2% for standard evaluation and 5.5 ± 14.0% for the new evaluation (P = ns). Evaluation time was significantly reduced from 11.6 ± 3.5 min to 7.6 ± 1.8 min for infarct size measurement (-33%, p < 0.001) as well for edema size measurement from 11.6 ± 3.2 min to 7.6 ± 1.7 min (-33%, p < 0.001).

## Conclusions

The new evaluation strategy results in similar infarct and edema sizes in patients with acute infarction in comparison to standard evaluation with good agreement between observers. Evaluation times were significantly reduced using the new algorithm.

## Funding

None.

